# Syn-CpG-Spacer: A Panel web app for synonymous recoding of viral genomes with CpG dinucleotides

**DOI:** 10.21105/joss.06332

**Published:** 2024-04-03

**Authors:** Aleksander Sulkowski, Clément Bouton, Chad Swanson

**Affiliations:** 1 Department of Infectious Diseases, King’s College London, London, United Kingdom

## Abstract

Vertebrate genomes contain lower than expected frequencies of the CpG dinucleotide. Consequently, many vertebrate viruses have evolved to mimic this composition, possibly in order to evade host antiviral defences (Greenbaum et al., 2008). For example, the antiviral protein ZAP binds CpGs in viral single stranded RNA with specific spacing requirements (Gonçalves-Carneiro et al., 2022), though CpGs are also likely depleted in viral genomes due to other selective pressures (Forni et al., 2023). Increasing CpG abundance by synonymous recoding could facilitate attenuation of viruses without compromising their epitope antigenicity by changing non-CpG codons to alternatives containing CpG without changing the overall amino acid sequence (Gonçalves-Carneiro et al., 2022; Le Nouën et al., 2019; Sharp et al., 2023). There are three ways CpGs can be synonymously introduced in codons: at positions 1-2 for arginine (e.g. AGA → CGA), 2-3 for several amino acids (e.g. ACA → ACG), or in a 3-1 split configuration, if a subsequent codon begins with a G (e.g. ATA-GCA → ATC-GCA).

Syn-CpG-Spacer is a Python progressive web app (PWA) (MDN Web Docs, 2023) made with the Panel library (Panel Development Team, 2024) that allows for consistent recoding of viral sequences and applying biologically relevant constraints. These include setting a minimum gap between CpG’s, optimising for an average CpG gap, protecting cis-acting regulatory signals from modification, and modulating the A-content in the overall sequence. The app features a sequence viewer made with the Bokeh library (Bokeh Development Team, 2024) that highlights CpG dinucleotides, allowing for efficient analysis of the resulting distribution of CpGs. This is complemented by a statistical data table. Utilising Biopython (Cock et al., 2009) modules, the user can load their sequence as a FASTA file and download the outputs as an alignment in the same format. As a PWA running on Pyodide (The Pyodide development team, 2023), the code is only executed in the user’s browser and they can install the app onto their machine for offline use.

## Statement of need

There are currently no published tools that allow specifying the spacing of CpG dinucleotides when synonymously recoding sequences. SSE can recode a sequence to a defined CpG frequency, and has a graphical interface (Simmonds, 2012). However, it cannot control the spacing of the CpGs. Newer tools require the knowledge of R, such as SynMut (Gu & Poon, 2023). A problem with bioinformatics packages is a high access barrier for users who are not familiar with programming languages. Here, the installation and update processes are streamlined thanks to the PWA approach.

With the current lack of CpG recoding tools, researchers may turn to in-house solutions which can hamper the reproducibility of their results, while also introducing the room for error. Syn-CpG-Spacer makes it more efficient to synonymously recode a sequence compared to doing so without any support tools.

## Research applications

In recent years, there has been an increased research focus on introducing CpGs into viral genomes as a mechanism to create live attenuated virus vaccines, such as influenza A virus (Gaunt et al., 2016; Sharp et al., 2023). However, the mechanism of action for how CpGs restrict the virus is unclear and it could be due to sensitising it to antiviral proteins such as ZAP (Ficarelli et al., 2021) or other poorly characterised effects on viral gene expression. This tool can be used to introduce CpGs into different viral genomes with specific spacing to determine if this attenuates the virus in vitro or in vivo and characterise the mechanism of attenuation, which will aid the development of live attenuated viral vaccines (Le Nouën et al., 2019).

Another potential application for the software is creation of CpG islands, which are long stretches of DNA rich in CpG dinucleotides that allow for epigenetic control of transcription. While most vertebrate CpGs are methylated, and thus transcriptionally silent, the DNA in CpG islands is hypomethylated, facilitating transcription factor binding (Angeloni & Bogdanovic, 2021).
